# Traffic police officers’ use of first aid skills at work: a qualitative content analysis of focus group discussions in Dar Es Salaam, Tanzania

**DOI:** 10.1186/s12873-020-00368-1

**Published:** 2020-09-10

**Authors:** Menti L. Ndile, Britt-Inger Saveman, Gift G. Lukumay, Dickson A. Mkoka, Anne H. Outwater, Susann Backteman-Erlanson

**Affiliations:** 1 Department of Clinical Nursing, Muhimbili University of Health and Allied Sciences (MUHAS), P.O. Box 65001, Dar es Salaam, Tanzania; 2 Department of Nursing, Umeå University, Umeå, Sweden; 3 Department of Community Nursing, MUHAS, Dar es Salaam, Tanzania

**Keywords:** Post-crash care, Training, Facilitators and hindrances, Traffic police

## Abstract

**Background:**

The World Health Organisation (WHO) recommends involving lay people in prehospital care. Several training programmes have been implemented to build lay responder first aid skills. Findings show that most programmes significantly improved participants’ first aid skills. However, there is a gap in knowledge of what factors influence the use of these skills in real situations. The current study aimed to describe police officers’ views on and experiences of factors that facilitate or hinder their use of trained first aid skills at work.

**Methods:**

Thirty-four police officers participated in five focus group discussions. A structured interview guide was used to collect data. Interviews were audio-recorded and transcribed verbatim. Data were analysed using qualitative content analysis.

**Results:**

We identified five categories of facilitators or hindrances. Training exposure was considered a facilitator; work situation and hospital atmosphere were considered hindrances; and the physical and social environments and the resources available for providing first aid could be either facilitators or hindrances.

**Conclusion:**

Practical exposure during training is perceived to improve police officers’ confidence in applying their first aid skills at work. However, contextual factors related to the working environment need to be addressed to promote this transfer of skills.

## Background

Emergency prehospital care in low- and middle-income countries is underdeveloped, due mostly to resource constraints [1, 2]. One important constraint is the lack of skilled people to provide first aid to road crash victims. In this situation, people with no medical background (lay responders) are responsible for the care of crash victims at the scene and on the way to the hospital [3]. The World Health Organization (WHO) recommends basic first aid training for potential lay responders, including police officers, to build their capacity to provide appropriate initial assistance before professional care providers take over responsibility [4].

In response to WHO recommendations, several lay-responder training programmes and models have been implemented and evaluated. Findings show that most of these programmes have significantly improved first aid knowledge and skills in participants [5–9]. However, the success of a training programme is determined by, among other things, the extent to which the learned skills are applied over time in the workplace [10]. Therefore, understanding the factors that influence the use of first aid skills at work is vital for programme scalability and sustainability. More context-based studies are needed to shed light in this area considering that only a few studies [6, 11, 12] of lay training in prehospital care have examined this aspect.

Among the few existing studies, one study attributed lack of time, resources and safety as barriers to apply first aid skills [6]. Another study regarding bystander cardiopulmonary resuscitation (CPR), linked low bystander CPR performance rates to poor quality of training related to lack of using realistic scenarios, lack of individual training times and lack of feedback from instructors [11]. Another study on CPR knowledge transfer conducted among older people described lack of self-confidence as a barrier to application of CPR skills [12].

Due to limited literature on factors influencing the transfer of trained first aid skills in the prehospital environment, the current study aimed to describe police officers’ views on and experiences of factors that facilitate or hinder their use of trained first aid skills at work in Tanzania. Police officers participated in this study because they are usually the first people to be called to attend the crash scene in urban Tanzania to provide assistance to road crash victims.

## Methods

### Study design and setting

The study used qualitative inductive content analysis of focus group discussions (FGDs). This study is a follow-up of a training programme for traffic police on providing Post crash First Aid (PFA), which was conducted as part of a large research project on injury prevention and care in Tanzania (INPACT). FGDs were used to gain a broad overview of trainees’ views and experiences of using their first aid skills [13]. The study was conducted in Dar es Salaam, the commercial capital of Tanzania, with an estimated population of more than 5.1 million [14].

### Post-crash first aid training

The educational training aimed to provide basic knowledge and skills to police officers about managing injured victims at a crash scene and on the way to the hospital. The researcher and members of the INPACT project developed a PFA course that aimed to addressing gaps found by the previous study conducted in Tanzania [15]. The study indicated that traffic police had little knowledge and practical skills to care road crash victims. The course was developed according to WHO guidelines on essential knowledge and skills for provision of basic prehospital care [4]. Three experienced emergency and trauma care teachers facilitated the course. Every traffic police officer attended a total of 16 h (2 days). About 27 traffic police officers attended the course per sitting. A total of 135 traffic police officers completed the course. Topics covered during the course were scene survey, provider safety, and initial assessment of injured victim. They were conducted as lectures and discussions involving the whole group of 27 traffic police officers. Practical training followed involving small groups of 8 to 10 police officers. Traffic police officers practiced on how to manage airway and breathing problems, control of external bleeding, care fractures of extremities, recovery positioning, and head and neck immobilization. A mannequin was used for demonstration and practice followed by role plays. At the end of the training, police officers were given reference materials on basic first aid care in form of leaflets [16].

### Study participants and recruitment

Police officers who completed a PFA training conducted 6 months earlier and had been at a crash scene since then were eligible to participate in the study. In total, 34 police officers (9 women and 25 men), aged between 27 to 54 agreed to participate in the study. The selection of police officers was based on four identified police jurisdictional areas. Due to their tight schedules, five different locations in respective jurisdictional areas were selected by police officers as convenient places for participating in FGDs. The required number of FGDs and participants were based on literature reviews indicating that 6 to 10 people are recommended participants in a single FGD and 3 to 6 FGDs are enough to provide trustworthy answers to research questions [13, 17, 18]. Five FGDs were conducted, the largest comprising 8 police officers (3 women and 5 men) and the smallest, 6 (1 woman and 5 men).

All participants were informed of the purpose of the study and were asked orally and in writing for their consent to participate in the study. The study was approved by the Institutional Review Body of Muhimbili University of Health and Allied Sciences (MUHAS).

### Data collection

The interview guide was developed, tested, and evaluated with 2 participants who were not included in the main study. Originally there were six topic questions, but because they were found to be repetitive and confusing for participants, we reduced them to three. FGDs were then conducted by the researchers, a moderator (MLN), and an assistant moderator (GGL). The interview guide comprised the following questions: 1) What is your experience concerning the application of acquired skills at your workplace following first aid training? 2) In your view what are the things/situations that enabled you to apply the skills acquired during training at your workplace? and 3) In your view, what are the things/situations that hindered you from applying the skills acquired during training at your workplace? Probe questions were asked to gain more understanding of important issues attached to the topic. See Additional file 1 for details.

The interviews were conducted in Swahili (average 47 min) and audiotaped**.** All interviews were transcribed verbatim in the original language prior to data analysis. Two of the five interviews were further translated into English to enable two co-authors not conversant in local language to follow the analytic process and take part in the forthcoming discussions.

#### Data analysis

Qualitative content analysis was performed [19]. The first author (MLN) read and re-read the interviews to get a sense of the whole. Meaning units were then identified, condensed, and labelled with codes while preserving the core meaning. All codes from the Swahili transcripts were translated into English. Codes with similar patterns were grouped into categories and subcategories. To ensure rigour and consistency in the interpretation of data, every step of the analysis was discussed with co-authors BIS and SBE; when disagreement arose, the process was repeated until consensus was reached. The first author also back-translated the subcategories and categories to Swahili to determine whether they matched the coded extracts from the interviews. The process was followed by DAM, who is fluent in both Swahili and English. See Table 1 for an example of data analysis process.

**Table 1:** Example of the analytic process

Meaning unit	Code	Subcategory	Category
‘We have our emergency number, any person can call, and then we receive the information from the station where the accident has occurred.’ (Participant in FG 1)	Emergency number	Emergency call system	Resources for first aid
‘I tell you we are not given gloves or anything as part of our job. If you love yourself, you will have to buy them, or when you send the victim to hospital you will privately ask a nurse to give you some*.*’ (Participant in FG 1)	Lack of gloves	Equipment and materials for first aid	
‘Many times, we ask for a lift from people or we take motorcycle taxis to get to the site of an accident because we have few vehicles.’ (Participant FG 5)	Inadequate police vehicles	Transport to and from the crash scene	

## Results

The analytic process resulted in five categories and 11 subcategories of facilitators or hindrances to using first aid skills at work (See Table 2). Examples of quotes extracted from the responses of police officers are provided in the text for each category and subcategory.

**Table 2:** Overview of categories and subcategories

Categories	Subcategories
Training exposure	Training methods
	Course content
Work situation	Decision making authority
	Staff shortage
Physical and social environment	Accessibility of the crash location
	Support from people around crash scene
Resources for first aid	Emergency call system
	Equipment and materials for first aid
	Transport to and from the crash scene
Hospital atmosphere	Referral and admission processes
	Attitude of healthcare providers

### Training exposure

This category involves two subcategories*: training methods* and *course content*. It describes how the the training facilitated the use of first aid skills on site.

#### Training methods

Hands-on practice significantly contributed to the ability of police officers to apply first aid skills more appropriately. They explained that before the first aid training they used to care for victims merely through guessing because their knowledge and skills were limited. They said that by reflecting on their training in first aid procedures during the course, they were easily able to apply their skills in real situations.

‘So now if something happens you remember what we did in the training and you do it properly. In that training we were told how to do things practically (through manikin and role plays), so you apply it in the real situation.’ (Participant in FGD 1)

#### Course content

Teaching trauma conditions similar to what police officers commonly encountered while on site was considered to boost police officers’ confidence in applying their skills they learned in class as they could easily familiarize with them. However, they confessed that at times care of real crash injury victims could be more overwhelming and challenging than those in a simulated environment.

‘We were called to an accident that happened. When we got there, I found that they had placed the victim on the back. After doing assessment the way you trained us, I realised she had lost consciousness. So, I placed her on her side.’ (Participant in FG 1)

### Work situation

This category refers to the time and space available for using first aid skills at the crash scene. Two subcategories were identified as barriers to using their first aid skills: *decision-making authority* and *staff shortage*.

#### Decision-making authority

Working with a senior officer who had no first aid education was treated as a barrier, because senior officers tended not to follow junior officers’ suggestion about the management of victims. In some situations, their decision to help was interrupted by a senior officer’s priorities; they had to follow orders and so were hindered in assessing the scene and the safety of the victim.

‘The boss tells you “Pick him/her up and let’s go”, so there is no time. And I have seen that as a challenge that we face when we attempt to care for the victim in our work.’ (Participant in FG 2)

#### Staff shortage

Opportunity to apply first aid skills was hindered by shortage of police officers. In addition they have to attend to other responsibilities such as to ensure smooth flow of traffic and legalities associated with the crash.

‘In our area a lot of accidents happen during the night. At that time only one officer manages the area, so when an accident happens, you don’t have time to attend to the victim, and even help from other people may not be available.’ (Participant in FG 3)

### Physical and social environment

This category refers to the physical and social environment where the crash occurred. Three subcategories are described as either facilitating or hindering application of first aid skills: *accessibility of the crash location* and *support from people around crash scene*.

#### Accessibility of the crash location

Ability of police officers to reach the crash location in time and provide meaningful interventions to victims was often limited by mobility issues. When the crash was far away from the police station, getting to the scene became a challenge, which meant they were routinely late in reaching the crash scene.

‘Because sometimes accidents happen far away from our position, for me to get there, the victim will already be in a very poor situation for me to do something.’ (Participant in FG 4)

#### Support from people around the crash scene

Presence of people around the crash scene was considered to facilitate provision of first aid by police officers. They described getting support from people in forms of manpower, materials, and moral support.

‘I would say that the thing that helped us to use our skills was the readiness of people around to work with me to help the victim, for example, helping me to lift a victim into a vehicle*.*’ (Participant in FG 3)

On the other hand, people’s ignorance and misconceptions about the care of victims creates mistrust and interference with care at the scene. This was described as a hindrance in the care process.

‘People think differently. While you have knelt down trying to assess the victim, others around think that you are wasting time, because they don’t know … so they start shouting. If it happens that the relatives of the victim are around, they may just decide to take matters into their own hands’*.* (Participant in FG 1)

### Resources for first aid

Care of injured victims requires the use of resources at the scene and on the way to the hospital. In this category, three subcategories were identified as facilitating or hindering the use of first aid skills: *emergency call system*, *equipment and materials for first aid*, and *transport to and from the crash scene*.

#### Emergency call system

Having an emergency telephone number available for people to call police about crashes facilitated police officers to reach the scene and provide help. They reported that people sometimes called them directly through their own mobile numbers or the public emergency call numbers.

‘For example, when an accident happens somewhere at Geza, I may not know until someone calls the police. So, when we get such information, we rush to the scene to provide any needed help.’ (Participant in FG 3)

#### Equipment and materials for first aid

Lack of equipment for first aid was reported by all police officers as a barrier to using their first aid skills. Police officers mentioned the lack of gloves for self-protection and boards for lifting and carrying injured victims as big challenges. They explained that they often found themselves in difficult situations, especially with victims who are bleeding.

‘You may go to the scene and find the victim is bleeding profusely and you don’t have gloves, bandages, or any piece of cloth. So, it is difficult to proceed with care.’ (Participant FG 3)

#### Transport to and from the crash scene

Inadequate transport in the police department was described as a challenge to officers’ ability to reach the crash scene on time and provide first aid. Not all police officers have vehicles; they often have to rely on getting a lift from other people or paying for motorcycle taxis to get them to the crash location. Lack of appropriate vehicles to transport victims to hospital was also a challenge to their ability to apply first aid during transport because of the confined space.

‘Even if you succeed in getting a vehicle, they are not suitable to transport victims because we use private cars and find it difficult to accommodate serious victims.’ (Participant in FG 3)

### Hospital atmosphere

This last category refers to the interaction between healthcare providers and police officers and the disposition of the victim at the hospital. Two subcategories were identified as discouraging police officers’ efforts to provide first aid care: *processes of referral and admission* and *attitude of healthcare providers*.

#### Processes of referral and admission

Admission to and referral procedures at hospitals were reported by police officers as lengthy and bureaucratic, this hindered their morale and ability to give good care to victims. For example, the police officers described that they could see that the condition of victim is deteriorating, but the victim had to stay at the hospital for hours just waiting for transfer to another hospital capable of providing needed treatment. This discouraged their putting more effort in care process as they thought that their efforts were wasted. Furthermore, it could happen that they were asked to pay some amount for admission bills for the victim to cover for initial costs while processes for identifying relatives of the victim are taken.

‘This is very bad because they saw I’m a police officer and that this was an emergency.’ (Participant in FG 3)

#### Attitude of healthcare providers

Slow action of healthcare providers at hospitals in taking responsibilities of patient care from police officers was viewed by police officers to hinder their morale to further engage in the care of victims. They described that having done their job of transportation as well as they could, but then being in the emergency department for almost an hour waiting for a doctor or a nurse to attend the victim.

‘When we bring victims, we find that nurses are not helpful. They just watch you. They should be quick in taking care of the victim*.*’ (Participant in FG 4)

## Discussion

This article aimed to describe police officers’ views on and experiences of factors that facilitate or hinder their use of trained first aid skills at work. Various issues were raised and are discussed in terms of the facilitators and barriers found in the categories *training exposure*, *work situation*, *physical and social environments*, *resources for first aid* and *hospital atmosphere*. Our main finding show that lack or inadequate resources such as equipment and materials needed for providing first aid was a major issue that prevented police officers to have an opportunity to use their trained first aid skills effectively.

Our study shows that hands-on learning during training improves learners’ confidence in using first aid skills in real settings. This was also demonstrated by a systematic review reporting that hands-on training generally increased trainees’ self-confidence and willingness to perform skills in real-life settings [20]. However, other factors in the training and working environments may significantly contribute to how learners’ confidence in using trained first aid skills is put into action.

The findings indicates that the work situation may influence motivation to provide first aid. For example, lack of individual decision-making authority in the care process has been shown to reduce opportunities to utilise one’s skills. In an organisation like the police force, where decisions are based mainly on hierarchy, rank seniority may outweigh other attributes in making decisions. For example, most police officers in our study who attended first aid training were junior officers. However, senior officers were in charge of managing crash scenes and injured victims regardless of their knowledge. Therefore decisions based on hierarchy rather than expertise and team spirit may not achieve the best patient outcomes [21]. Moreover, other work-related responsibilities may overwhelm officers with first aid training and reduce their opportunities to perform first aid. Similar situations were also reported in a Ugandan study on the effectiveness of a lay-responder prehospital training programme [6].

Physical and social environments may positively or negatively influence engagement in first aid care. Difficulty in accessing a crash was described as a hindrance to police officers trying to provide immediate care. First responders difficulty in accessing a crash site was also reported in previous studies [22, 23]. Furthermore the tension and anxiety from crowds at the crash scene made the environment uncomfortable for those providing care, as also reported in previous studies [22, 24, 25]. On the other hand, however, our study showed that people around the crash scene could sometimes be helpful to first responders when there was a good communication on both sides.

In the context of Tanzania, where no medical dispatch centres exist, bystanders usually call police officers to report crashes. Police officers in our study found such bystander calls to facilitate their arrival at the crash location. Quantitative studies are needed to understand the level of awareness, use of emergency call numbers, and effectiveness of calls in facilitating first aid intervention.

Lack of, or inadequate, resources may also prevent police officers from providing first aid care. For example, a lack of gloves may prevent bleeding control out of the provider’s fear of becoming infected. A similar finding was reported in a study involving participants who are not police officers [26]. Lack of first aid resources create a dilemma in the management of trauma victims as the intention to intervene conflicts with personal safety concerns. This may ultimately lead to withholding care. Lack of reliable and appropriate transport to and from the crash scene was another barrier that affected timely and proper first aid care. Due to inadequate ambulances, the vehicles of passing motorists are often expropriated, complicating victim care because the vehicles lack adequate space and basic equipment. Similar transportation concerns have been previously reported [27–29].

Our participants reported unencouraging hospital atmosphere as a potential barrier to their using first aid skills and a drain on their morale. According to the WHO’s essential trauma care guideline, trauma victims must be stabilised at the first hospital before being referred to higher hospitals for definitive care [30]. Unfortunately, significant number of patients in Tanzania do not receive this care and may wait several hours before being referred to an advanced hospital [31]. This can discourage responders from fully engaging in care. They often think that their efforts are wasted by complicated and lengthy admission processes, particularly when a trauma victim has no known relatives to cover the treatment and associated costs. Moreover poor communications and lack of role clarity were perceived by responders to contribute to poor interactions with emergency department healthcare providers. This calls for establishment of strategies to improve good cooperation between first responders and healthcare providers at hospitals.

### Implications for prehospital care practice

Findings from this study show how issues related to physical, social, resource and work system influence the opportunity of police officers to use their trained first aid skills in the prehospital environment. We suggest that these issues are addressed by relevant authorities so as to facilitate the use of first aid skills in settings with similar challenges. In a police force were implementation of most decisions are influenced by personal rank or seniority, we suggest that both senior and junior police officers participate on-the –job first-aid training programmes, this may improve teamwork and care given to road crash victims. We further suggest regular meetings between police officers and healthcare providers in various platforms to address mutual challenges in order to improve care of road crash victims.

### Limitation of the study

The current study is based on police officers’ views and experiences of using their first aid skills at work. Other people such as drivers may be involved in providing initial assistance to trauma victims, and their views may differ with those of the police officers in explaining prehospital care challenges.

## Conclusion

Practical exposure during training is perceived to improve police officers’ confidence in applying their first aid skills at work. However, contextual factors related to the working environment need to be addressed to promote this transfer of skills.

## Data Availability

The data set generated and/or analysed during the present study is not publicly available due to the confidential nature of the data, but is available from the corresponding author on reasonable request.

## Abbreviations

CPR: cardio-pulmonary resuscitation
FG: focus group
FGD: focus group discussion
MUHAS: Muhimbili University of Health and Allied Science
PFA: post-crash first aid
WHO: World Health Organization
